# A case report of a patient with severe thyroid eye disease

**DOI:** 10.22336/rjo.2022.30

**Published:** 2022

**Authors:** Mioara-Laura Macovei, Űnal Azis

**Affiliations:** * Alcor Clinic, Bucharest, Romania

**Keywords:** Graves’ disease, exophthalmos, corneal ulceration

## Abstract

**Objective:** Our aim was to present a rare case of a middle-aged male patient, diagnosed with Graves’ orbitopathy, which had an atypical rapid unilateral onset. Initially, the left eye presented exophthalmos, eyelid retraction, corneal ulceration, and pannus formation with an important vascular component due to corneal exposure. The same symptoms developed in the right eye within a short period of time.

**Methods:** A 52-year-old man presented in our department with bilateral proptosis, decrease in visual acuity, and orbital pain, which developed initially in the left eye seven months before the right eye. Slit lamp examination revealed conjunctival hyperemia, purulent discharge, chemosis and inflammation of the caruncle in both eyes. The fluorescein eye stain test was positive due to corneal ulceration with the presence of cells and flare in anterior chamber in the RE (right eye). The LE (left eye) presented a corneal pannus. We documented the changes using a slit lamp biomicroscope, a fundus camera, orbital ultrasonography, and contrast CT (computer tomography) scans.

**Discussions:** The severe Graves’ ophthalmopathy represents a challenge both in active or inactive phase. Medical and surgical therapies should be taken into consideration in order to prevent the complications following corneal perforation or optic neuropathy. Also, ophthalmic, and systemic adverse reactions of systemic steroids used in the treatment of Graves’ disease are important in the prognosis of the visual outcome.

**Conclusions:** The management of Graves’ ophthalmopathy is multidisciplinary and needs a very good therapy adherence in order to achieve a satisfactory prognosis and quality of life.

## Introduction

Thyroid associated orbitopathy (TAO) represents an autoimmune, self-limiting disease that occurs in patients with Graves’ disease (90%), Hashimoto thyroiditis (3%), primary hypothyroidism (1%) or in the absence of thyroid abnormalities (6%) [**1**].

It is known as the most common orbital disease globally, which has a peak of incidence between fourth and sixth decade. The risk factors for developing TAO are represented by female gender, smokers, family history, life stressors and poorly controlled hypothyroidism following radioactive iodine, whereas male gender, increasing age and a rapid onset of orbitopathy are the predictors of severe thyroid eye disease [**2**]. The target cells of the immune process are represented by the pluripotential orbital fibrocytes, which are both found in orbital fat tissue and striated muscle. TRAB (thyrotropin-receptor antibodies), also known as TSH-R (thyroid stimulating hormone receptor) are secreted by an aberrant population of lymphocytes in Graves’ disease. The circulating TRAB binds to CD40+ receptors expressed by the orbital fibrocytes and induces the upregulation of inflammatory cytokines such as IL-6, IL-8, and PGE E2, which increases the production of hyaluronan and glycosaminoglycan. The expansion of orbital fat is caused by a subpopulation of fibrocytes, which differentiates into adipocytes. The clinical features depend on the orbital tissue involved [**2**]. The most common symptoms and signs of TAO are upper eyelid retraction (75%), lid lag with downgaze (50%), and dull orbital pain (35%). Most TAO patients develop a pattern that involves fat expansion, has a slow progression, and occurs predominantly in young females. This pattern is represented by eyelid retraction, proptosis, and ocular exposure [**1**]. The muscle centric pattern affects older people, has an increased severity and it is highly associated with smoking and family history of TAO. The enlargement of the extraocular muscles determines restricted ocular ductions, diplopia, edema and congestion of eyelids and conjunctiva and dysthyroid optic neuropathy [**1**].

## Case presentation

**Fig. 1:**
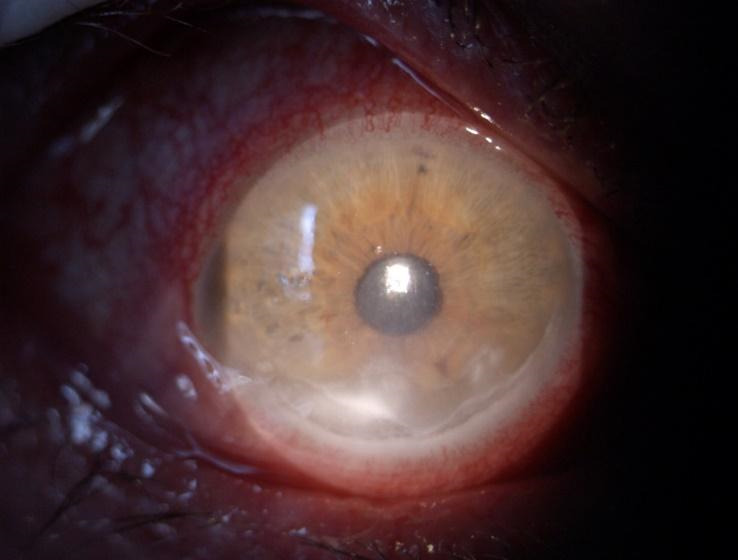
RE: Corneal inferior ulceration with KPs and important hyperemia

**Fig. 2:**
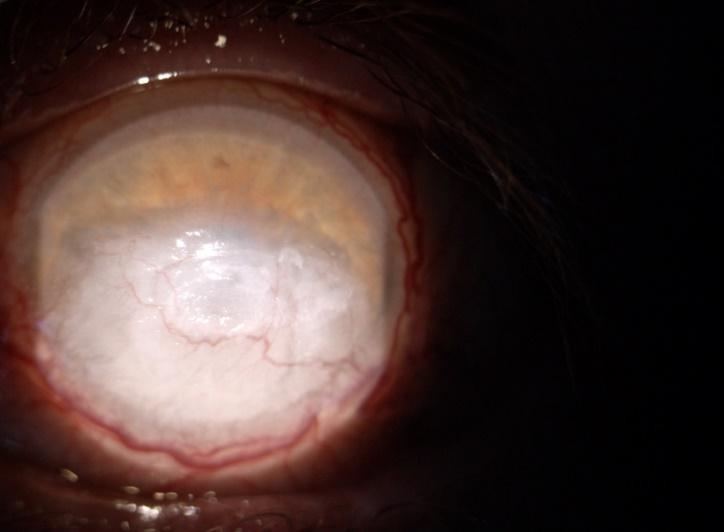
LE: Corneal pannus with vascularization

**Fig. 3:**
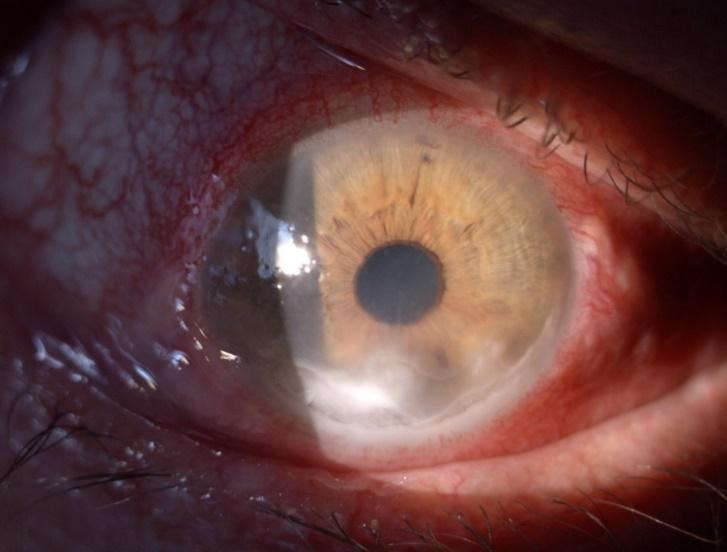
Right eye (RE) at presentation

A 52-year-old Caucasian male was referred to our Ophthalmology Department for a one-month history of bilateral orbital pain, proptosis and decrease in visual acuity. The patient reported that the onset of symptoms was unilateral, initially affecting the left eye, for which he was diagnosed six months before with preseptal cellulitis in another Ophthalmology Service and was treated with systemic and topical antibiotics, non-steroidal anti-inflammatories, mydriatics and preservative free lubricants. The symptoms did not improve after the initial treatment. No family history of thyroid disease was known, nor previous exposure to radioactive iodine. The patient reported a recent decrease in visual acuity of the right eye with blurring for approximately one month, associated with anorexia, chronic headache, and fatigability. He was also a heavy smoker (60 cigarettes per day for more than 20 years). Physical examination showed bilateral marked proptosis (24 mm in RE, 26 mm in LE), mainly affecting the left eye, eyelid retraction, erythema and edema of the eyelids and low-grade orbital pain on palpation. The best corrected visual acuity was 0.6 on RE and the patient perceived the hand movements with the LE. The intraocular pressure was normal (12 mm Hg) in RE and increased on palpation in the LE. Ophthalmological examination revealed conjunctival redness and purulent discharge, chemosis, inflammation of caruncles, evident restriction of the movement in both eyes. An inferior marginal corneal ulceration, cells, and flare in the anterior chamber and keratic precipitates on the posterior surface of the cornea were documented in the RE (**Fig. 1**). A corneal pannus affected the two-thirds of the cornea of the LE with the presence of dilated, tortuous vessels (**Fig. 2**). Fundus evaluation of the right eye was normal, without suggestive signs for dysthyroid optic neuropathy, whereas the left eye could not be evaluated. Ultrasound was performed and revealed the enlargement of the extraocular muscles, without other particular signs. According to the European Group on Graves’ Orbitopathy (EUGOGO) TED (thyroid eye disease) severity scale, the patient was referred with “very serious disease”, due to corneal ulceration and had a clinical activity score (CAS) of 7 at presentation (**Fig. 3**). The nervous system and systemic evaluation were normal.

The patient was admitted to hospital for further investigations, endocrinologic assessment and management. Initial blood investigations, including blood count, liver biochemistry, glycemia, serum electrolytes, fasting lipid profile, serum creatinine, blood urea nitrogen, erythrocyte sedimentation rate, C reactive protein were normal. The thyroid function and antibody tests were also performed, revealing hyperthyroidism based on high levels of FT3 and FT4, with low TSH. The presence of high levels of TSH-R autoantibodies suggested an active phase of Graves’ disease. Orbital imaging confirmed the diagnosis based on contrast CT scans, which showed enhancement of the extraocular muscle sheaths and stranding of surrounding orbital fat, typical findings for the active inflammatory phase. Cerebral MRI (magnetic resonance imaging) confirmed the absence of dysthyroid optic neuropathy or the presence of other lesions. The ultrasound exam of thyroid gland revealed: mixed echogenicity, diffusely enlarged thyroid gland, inhomogeneous structure and ventrally hypoechoic micronodular structure. Following the endocrinologic assessment and ophthalmic examination, the diagnosis of severe Graves’ ophthalmopathy was established.

The patient was hospitalized for two weeks. A protocol with intravenous methylprednisolone 500 mg weekly for 6 weeks, reduced to 250 mg weekly for 6 weeks was initiated. Topical broad-spectrum antibiotics were instilled in both eyes. Preservative-free lubricants were used in order to relief the symptoms of corneal exposure and a mydriatic was administered 3 times per day in the right eye in order to avoid the formation of posterior synechiae. The IOP of the LE was lowered by using a topical combination of timolol and dorzolamide. Doxycycline was also administered due to its efficiency in the treatment of inflammatory ocular conditions by inhibiting the synthesis and the release of corneal MMPs [**3**].

Patching of both eyes during night was also performed. Thyrozol and propranolol were prescribed to optimize the thyroid function. The patient was also instructed to cease smoking. A target in the management of Graves’ ophthalmopathy is to reduce the modifiable risk factors such as smoking. The patient received effective support in order to cease smoking. A clinical study revealed that cigarette smoking may corelate with increase in retrobulbar venous congestion in thyroid associated ophthalmopathy [**4**].

**Fig. 4:**
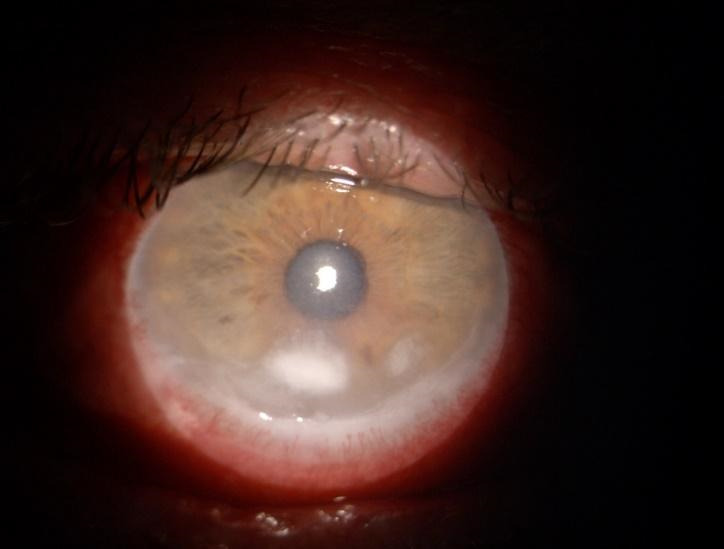
RE: Slight improvement of the corneal ulceration

A regular follow-up of the thyroid function at four weeks depending on the severity of the disease [**5**] and an ophthalmologic assessment was recommended by the endocrinologist. At the first month follow-up, the patient showed a suboptimal response to high dose intravenous methylprednisolone. We observed an increase of the CAS score suggested by the exophthalmos, which was similar to the previous examination, a decrease of best corrected visual acuity in RE and perception of hand movements in the LE, while the corneal ulceration showed a slight amelioration (**Fig. 4**). The thyroid function test showed euthyroidism. The liver and renal function were assessed and both were normal. The endocrinologist decided to continue the corticosteroid therapy in order to cease the inflammation. The radiotherapy or decompression surgery were not suitable in this case.

**Fig. 5:**
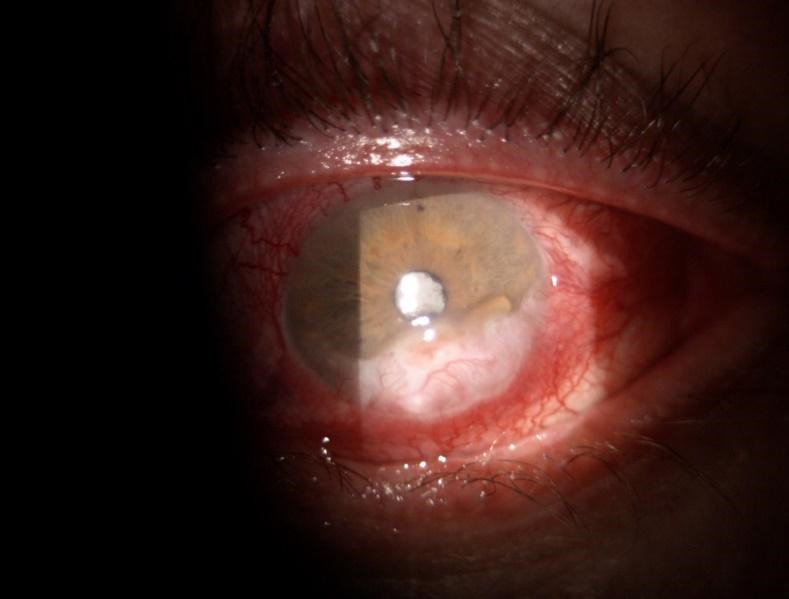
RE: Pannus formation and secondary cataract with synechiae

At the sixth month follow-up, the ophthalmologic examination revealed an improvement of exophthalmos in both eyes and a corneal pannus in RE similar to LE (**Fig. 5**). The visual acuity in RE showed an important decrease caused by a cataract, which developed secondary to systemic corticosteroid therapy. The thyroid test function revealed a drug-induced hypothyroidism and the treatment was switched to thyroid hormones substitutes. The patient delayed the surgical treatment of the cataract. The following ophthalmologic and endocrinologic exams did not reveal any modification of his status. 

## Discussion

The thyroid associated ophthalmopathy has a biphasic evolution, which was described by Rundle: an active, dynamic phase, which has a mean duration of 6-18 months [**1**] and an inactive, static phase [**6**]. In the dynamic phase, the immunomodulatory treatment and external beam radiotherapy are recommended in order to cease inflammation, while in the static phase, reconstructive surgery can be attempted [**1**].

The management of moderate-to-severe Graves’ ophthalmopathy consists of the control of the risk factors, local treatments, and immunosuppression [**7**].

A recent study concerning the trends in the treatment of active thyroid associated ophthalmopathy concludes that aggressive therapies such as oral or IV glucocorticoids, Rituximab and/ or Tocilizumab and orbital radiotherapy are used in the severe forms [**8**]. IV glucocorticoids in association with mycophenolate mofetil represent the first line therapy, while the oral prednisone or prednisolone with Azathioprine or Cyclosporine, Rituximab, Tocilizumab, and orbital radiation with oral or IV glucocorticoids are the second line therapy [**7**]. 

A novel IGF-1R (insulin growth-factor-1 receptor) antibody, Teprotumumab, plays an important role in the treatment of active Graves’ ophthalmopathy [**9**]. Also, in chronic phase, it showed benefits on proptosis, inflammation, diplopia, strabismus, and orbital soft tissue volume [**10**].

Moreover, topical steroids are prescribed in order to manage the symptoms of ocular inflammation consisting of dryness and hyperemia. Likewise, the ocular surface lubricating therapies are an important tool in the treatment of the exposure keratopathy [**8**].

The corneal involvement could be related to inflammatory processes including the systemic disease itself and the dry eye syndrome (DES) [**11**]. DES is common even in the early phases of Graves’ ophthalmopathy without exophthalmos. In Graves’ orbitopathy, DES is mainly evaporative due to corneal exposure and it can also be associated with CAS and reduced corneal sensitivity [**12**]. The alterations of the lacrimal film of these patients are caused by the impairment of the extraocular muscles and the immune-mediated dysfunction of the lacrimal glands [**13**].

The severe exposure keratopathy represents an urgence, thus decompression surgery could be indicated. In the active phase, local treatment such as tarsorrhaphies, corneal patches, or gluing can be used in order to avoid corneal perforation or to treat the corneal breakdown, while in the inactive phase, decompression, ophthalmic plastic, and strabismus surgery can be an important tool for the repair of residual damage (exophthalmos, lid retractions, eyelid, and periorbital puffiness, strabismus) [**7**].

## Conclusions

In conclusion, severe forms of Graves’ ophthalmopathy are sight-threatening and need a multidisciplinary approach of the management of the disease and its complications. Also, the therapy adherence and modifiable risk factors, such as smoking, play a major role for the outcome of the Graves’ ophthalmopathy.

**Conflict of Interest Statement**

The authors state no conflict of interest. 

**Informed Consent and Human and Animal Rights statement**

Informed consent has been obtained from the patient included in the study.

**Authorization for the use of human subjects**

Ethical approval: The research related to human use complies with all the relevant national regulations, institutional policies, it is in accordance with the tenets of the Helsinki Declaration and has been approved by the review board of Department of Ophthalmology, “Carol Davila” Central Military University Hospital, Bucharest, Romania.

**Acknowledgements**

None. 

**Sources of Funding**

None. 

**Disclosures**

None. 

**Contribution**

Both authors contributed equally to this article.
